# Impact of rewarming rate on interleukin-6 levels in patients with shockable cardiac arrest receiving targeted temperature management at 33 °C: the ISOCRATE pilot randomized controlled trial

**DOI:** 10.1186/s13054-021-03842-9

**Published:** 2021-12-17

**Authors:** Jean-Baptiste Lascarrou, Elie Guichard, Jean Reignier, Amélie Le Gouge, Caroline Pouplet, Stéphanie Martin, Jean-Claude Lacherade, Gwenhael Colin, AfterROSC network, M. Azais, K. Bachoumas, A. Bailly, L. Camous, G. Colin, L. Crosby, M. Fiancette, M. Henry Lagarrigue, J. C. Lacherade, J. B. Lascarrou, C. Lebert, L. Martin Lefevre, C. Pouplet, J. Reignier, A. Seguin, I. Vinatier, A. Yehia

**Affiliations:** 1 Médecine Intensive Reanimation, University Hospital Center, 30 Boulevard Jean Monnet, 44093 Nantes Cedex 1, France; 2 Paris Cardiovascular Research Center, INSERM U970, Paris, France; 3 AfterROSC Network, Paris, France; 4 INSERM CIC1415, CHRU de Tours, Tours, France; 5 Médecine Intensive Reanimation, District Hospital Center, La Roche-sur-Yon, France; 6 AfterROSC Network, Cochin University Hospital Center, Paris, France; 7 Medical Intensive Care Unit, District Hospital Center, La Roche-sur-Yon, France

**Keywords:** Cardiac arrest, Targeted temperature management, Therapeutic hypothermia

## Abstract

**Purpose:**

While targeted temperature management (TTM) has been recommended in patients with shockable cardiac arrest (CA) and suggested in patients with non-shockable rhythms, few data exist regarding the impact of the rewarming rate on systemic inflammation. We compared serum levels of the proinflammatory cytokine interleukin-6 (IL6) measured with two rewarming rates after TTM at 33 °C in patients with shockable out-of-hospital cardiac arrest (OHCA).

**Methods:**

ISOCRATE was a single-center randomized controlled trial comparing rewarming at 0.50 °C/h versus 0.25 °C/h in patients coma after shockable OHCA in 2016–2020. The primary outcome was serum IL6 level 24–48 h after reaching 33 °C. Secondary outcomes included the day-90 Cerebral Performance Category (CPC) and the 48-h serum neurofilament light-chain (NF-L) level.

**Results:**

We randomized 50 patients. The median IL6 area-under-the-curve was similar between the two groups (12,389 [7256–37,200] vs. 8859 [6825–18,088] pg/mL h; *P* = 0.55). No significant difference was noted in proportions of patients with favorable day-90 CPC scores (13/25 patients at 0.25 °C/h (52.0%; 95% CI 31.3–72.2%) and 13/25 patients at 0.50 °C/h (52.0%; 95% CI 31.3–72.2%; *P* = 0.99)). Median NF-L levels were not significantly different between the 0.25 °C/h and 0.50 °C/h groups (76.0 pg mL, [25.5–3074.0] vs. 192 pg mL, [33.6–4199.0]; *P* = 0.43; respectively).

**Conclusion:**

In our RCT, rewarming from 33 °C at 0.25 °C/h, compared to 0.50 °C/h, did not decrease the serum IL6 level after shockable CA. Further RCTs are needed to better define the optimal TTM strategy for patients with CA.

*Trial registration* ClinicalTrials.gov, NCT02555254. Registered September 14, 2015.

**Take-Home Message**: Rewarming at a rate of 0.25 °C/h, compared to 0.50 °C, did not result in lower serum IL6 levels after achievement of hypothermia at 33 °C in patients who remained comatose after shockable cardiac arrest. No associations were found between the slower rewarming rate and day-90 functional outcomes or mortality.

**140-character Tweet**: Rewarming at 0.25 °C versus 0.50 °C did not decrease serum IL6 levels after hypothermia at 33 °C in patients comatose after shockable cardiac arrest.

**Supplementary Information:**

The online version contains supplementary material available at 10.1186/s13054-021-03842-9.

## Introduction

The 2021 American Heart Association and European Resuscitation Council guidelines recommend: “actively preventing fever by targeting a temperature ≤ 37.5 °C for those patients who remain comatose after cardiac arrest followed by the return of spontaneous circulation (ROSC). Whether subpopulations of cardiac arrest patients may benefit from targeting hypothermia at 32–34 °C remains uncertain”. Moreover, the use of targeted temperature management (TTM) remains variable [1, 2], probably due in part to the paucity or poor quality of data on TTM strategies [3, 4]. That studies have produced conflicting efficacy data may be related to differences in various TTM components including rewarming parameters. Rewarming may be associated with myocardial function deterioration, a mismatch in oxygen consumption vs. delivery and, possibly, secondary neurological insults [5]. The most clinically significant rewarming parameter may be the rate of the temperature increase, which was 0.25–0.50 °C/h in the TTM1 and TTM2 trials [6, 7] and 0.4 °C/h and 0.66 °C/h in two other trials, respectively [8, 9]. To date, no randomized controlled trials (RCTs) have compared different rewarming rates.

The main goal of TTM at 32–36 °C in patients with coma after cardiac arrest is prevention of postcardiac arrest syndrome (PCAS) [6, 7]. A major feature of PCAS is systemic inflammation, of which interleukin-6 (IL6) is a key mediator. A post hoc analysis of data from the TTM1 trial [10] demonstrated significant IL6 elevation 24 h after cardiac arrest and a significant association between higher IL6 levels and greater severity of PCAS in both temperature groups (33 °C and 36 °C) [11]. Similarly, in two prospective observational studies of patients with out-of-hospital cardiac arrest (OHCA), IL6 elevation at ICU admission was independently associated with poorer functional outcomes [12, 13]. Thus, IL6 can probably serve as a marker for the severity of PCAS. Importantly, a study of multiple cytokines in 10 patients who were comatose after cardiac arrest suggested that hypothermia (32–34 °C) might not prevent the systemic inflammatory response and that rewarming might be associated with a proinflammatory effect [14]. Additional data on the effects of TTM parameters, notably the rewarming rate, on inflammation and IL6 levels are needed.

The objective of this single-center pilot RCT in patients managed with hypothermia at 33 °C for coma after OHCA in a shockable rhythm was to assess whether, compared to rewarming at 0.5 °C/h, slower rewarming at 0.25 °C/h was associated with lower serum IL6 levels.

## Methods

The study was registered on ClinicalTrial.gov (NCT02555254) on September 14, 2015.

### Trial design

The ISOCRATe (Impact of Speed Of rewarming after CaRdiac ArresT(e)) trial was an investigator-initiated, blinded-outcome-assessor, parallel-group, two-arm, pragmatic, single-center, RCT conducted in a medical-surgical ICU in France. The research protocol was approved by the appropriate ethic committee (*Comité de Protection des Personnes Ouest 3*, N°15-03-11). Written informed consent was obtained from the next of kin. When no next of kin was available, in compliance with French law, patients were included, informed of the study when they regained decision-making competency, and asked whether they wanted to remain in the trial; data from patients who requested full withdrawal were excluded from the analysis.

### Patients

Eligible patients were adults (18 years of age or older) who were unconscious (Glasgow Coma Scale [GCS] score ≤ 8 at ICU admission) after OHCA in a shockable rhythm, due to any cause, and who received TTM at 33 °C with a closed-loop cooling device (Artic Sun, BD, Montigny le Bretonneux, France). In patients who were already sedated at ICU admission, the GCS score determined by the emergency physician just before sedative therapy initiation was used. Exclusion criteria were as follows: no-flow time (from collapse to initiation of external cardiac massage) > 10 min; low-flow time (from initiation of external cardiac massage to ROSC) > 60 min; major hemodynamic instability (defined as a continuous epinephrine or norepinephrine infusion at a flow rate > 1 µg/Kg/min); time from cardiac arrest to study inclusion > 300 min; moribund patient (patient deemed by the attending physician to be likely to die before the end of the TTM maintenance phase, i.e., before randomization); Child–Pugh C cirrhosis of the liver; treatment with an IL6 receptor antagonist (e.g., tocilizumab); treatment with > 5 mg prednisolone-equivalent at the time of cardiac arrest; age < 18 years; pregnancy or breastfeeding; correctional facility inmate; previous inclusion in another RCT on cardiac arrest with day-90 functional outcome as the primary endpoint; no coverage by the statutory French health insurance system; and refusal by next of kin.

### Randomization and blinding

Patients were included at ICU admission. Randomization occurred at the end of the hypothermia maintenance phase, i.e., 18–24 h after reaching 33 °C (Additional file 1). This delay in randomization ensured that patients who died during hypothermia induction or maintenance, and who therefore had no IL6 data, were not included.

Patients were randomly allocated in a 1:1 ratio to either a 0.25 °C/h or 0.50 °C/h rewarming rate, using a web-based system accessible 24 h a day. The randomization sequence was generated by the statistician (ALG, who was not involved in patient recruitment), with permuted blocks of varying sizes and without stratification.

Blinding of the staff providing patient care was not feasible. However, the psychologist who assessed the day-90 outcomes in all patients was blinded to group assignment.

### Targeted temperature management (TTM)

The study protocol involved standardization of several parameters including sedation, neuromuscular blockade [15], and the management of expected adverse events. A closed-loop cooling device was applied immediately after ICU admission (ArticSun). The target temperature was 33 °C, which was to be achieved as rapidly as possible then maintained for 24 h. Body temperature was monitored using an esophageal probe [16].

#### Concomitant medications/treatments in both groups

- *Sedation*
  In both groups, all patients received sedation with midazolam combined with sufentanil according to the standard sedation protocol. Doses were adjusted to obtain a Richmond Agitation Sedation Scale score of -5 and were tapered when body temperature rose above 36 °C during the rewarming phase.
- *Shivering and neuromuscular blockade*

Persistent shivering was treated according to a previously published three-step protocol [15], which was adapted from the bedside shivering assessment score (BSAS). The goal was BSAS ≤ 1. Step 1 was administration of a single intravenous bolus of a hypnotic agent and an opioid, in doses equal to the hourly infusion rates of hypnotic and opioid drugs (i.e., 5-mg intravenous midazolam bolus if the continuous midazolam infusion rate was 5 mg/h). In step 2, an intravenous bolus of a nondepolarizing neuromuscular blocker (i.e., 10 mg of cisatracurium) was given. Step 3 consisted of a continuous infusion of the same nondepolarizing neuromuscular blocker (i.e., cisatracurium in an initial dose of 10 mg/h) to achieve BSAS ≤ 1; during rewarming, the infusion was stopped when the core body temperature increased above 35 °C.

#### Intervention group: 0.25 °C/h rewarming rate

The rewarming rate on the closed-loop cooling device was set at 0.25 °C/h followed by maintenance at 37 °C for 24 h.

#### Control group: 0.50 °C/h rewarming rate

The rewarming rate on the closed-loop cooling device was set at 0.50 °C/h followed by maintenance at 37 °C for 24 h.

### Functional prognostication and life-support withdrawal

All decisions to withdraw life-sustaining treatments were made according to current guidelines [6]. The decision-making process included routine evaluations of the functional prognosis based on multimodal parameters [7]. According to local policies, the intensivist in charge could obtain advice about the functional prognosis from an independent consultant.

### Follow-up and outcomes

The primary outcome was serum IL6 between 24 and 48 h after reaching the target temperature of 33 °C. Serum IL6 was assayed at achievement of 33 °C (H0) then 12, 24, 36, 40, and 48 h later, in both groups (Additional file 1). Only IL6 levels measured after randomization were considered for the analysis.

The secondary outcomes included the proportion of patients with a favorable functional outcome on day 90, defined as a cerebral performance category (CPC) score of 1 or 2. The CPC scale ranges from 1 to 5, with 1 indicating good performance or minor disability, 2 moderate disability, 3 severe disability, 4 coma or vegetative state, and 5 brain death. The CPC was assessed during a semi-structured telephone interview by a single psychologist specifically trained for the study and blinded to the treatment group. Other secondary outcomes were ICU mortality, day-90 mortality, ICU stay length, and duration of mechanical ventilation (MV). At the same timepoints as for IL6, the following serum biomarkers were assayed: IL2, IL4, IL8, IL10, granulocyte–macrophage colony-stimulating factor (GM-CSF), tumor necrosis factor alpha (TNFα), C-reactive protein (CRP), and procalcitonin. The values were compared between the two groups, as well as serum neurofilament light chain (NF-L) assayed 48 h, and serum neuron-specific enolase (NSE) 48 h and 72 h, after the OHCA.

A Luminex Magpix-based assay (Procartaplex kit, ThermoFisher Scientific, Courtabœuf, France) was used to measure serum IL2, IL4, IL6, IL8, IL10, granulocyte–macrophage colony-stimulating factor (GM-CSF), and TNFα, with the Ella™ instrument calibrated using the in‐cartridge factory standard curve, according to the manufacturer’s instructions. Serum NF-L was assayed using the Ella Simple Plex™ system (Protein Simple kit, Bio-Techne, Rennes, France) according to the manufacturer’s instructions [17].

### Sample size

The sample size was estimated as recommended by others for outcomes consisting in repeated measures [18, 19]. We expected that the mean IL6 level at 48 h would be 100 pg/mL in the 0.25 °C/h group and 15% higher, i.e., 115 pg/mL, in the 0.50 °C/h group, based on an earlier study [14]. We assumed a correlation of 0.85 between two IL6 values in a given patient, which was less accurate than the 0.89 correlation estimated by the assay manufacturer. Assuming a standard deviation of 40 pg/mL for the mean IL6 value, with the Type I error set at 0.05 and the Type II error set at 0.10, 25 patients were required in each arm to detect a 15 pg/mL difference between the two study arms. We expected about 20% of patients to die from refractory shock before randomization [20] and therefore planned to include 30 patients in each arm. This method complied with published information on sample size estimation for pilot trials [21].

### Statistical analysis

Categorical variables were described as number (percentage) and continuous variables as median [interquartile range].

To assess the intervention effect on the primary outcome, we used the trapezoidal rule to estimate the areas under the curves (AUCs) of serum IL6 levels 24–48 h after reaching 33 °C, and we compared these AUCs between the two groups by applying Wilcoxon’s rank test. Median IL6 levels at each time point were also compared using Wilcoxon’s rank test.

The primary outcome defined on ClinicalTrials.gov (NCT02555254) was measured between H0 (achievement of 33 °C) and H48, but patients were randomized between H18 and H24 (initiation of rewarming). We did not compare IL6 levels before randomization, in accordance with recommendations about RCTs [22].

Serum assays of biomarkers that were secondary outcomes (i.e., IL2, IL4, IL8, GM-CSF, and TNFα) were dichotomized based on the detection threshold, reported as *n* (%), and compared between the two groups using Fisher’s exact test. Continuous secondary outcomes were described as median [interquartile range] and compared using Wilcoxon’s rank test. Secondary outcomes reported as cumulative incidences were analysed using the competing risks approach, with ICU discharge and hospital discharge as the competing risks.

Clinical baseline characteristics regarding coronary revascularization, and clinical events qualified as adverse events were added as post hoc analysis on reviewer request without statistical testing performed.

No imputation strategy for missing data was used. SAS version 9.4 (SAS Institute, Cary, NC) and R version 3.3.1 were used for the statistical analyses. For this pilot exploratory trial, we did not adjust *P* values for multiple testing [23]. Therefore, the results for the secondary outcomes should be interpreted as exploratory.

## Results

### Patients

**Fig. 1:**
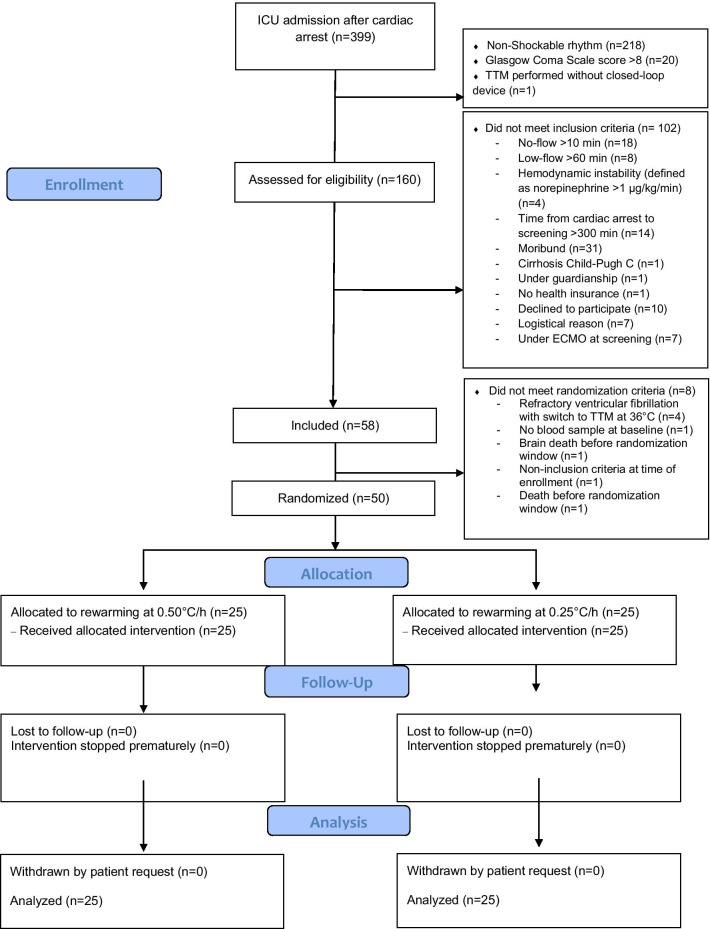
Patient flowchart

Of 399 patients assessed for eligibility between February 12, 2016, and May 15, 2020, 58 were included and 50 were randomized. No patient withdrew consent (Fig. 1). A descriptive comparison of included but not randomized vs. included and randomized patients is provided in Additional file 2. Table 1 lists the main baseline characteristics of the randomized patients.

**Table 1:** Baseline characteristics of the study participants

	0.25 °C/h rewarming rate (n_1_ = 25)	0.50 °C/h rewarming rate (n_2_ = 25)
Age (years)	66.2 [52.2; 72.4]	53.8 [48.2; 70.0]
Male sex	21 (84.0)	20 (80.0)
Charlson comorbidity index^a^	3.0 [1.0; 4.0]	1.0 [0.0; 3.0]
McCabe score, *n* (%)		
Disease expected to become fatal within 5 y	6 (24.0)	3 (12.0)
Disease expected to be fatal within 1 y	0 (0.0)	0 (0.0)
No fatal disease or unknown	19 (76.0)	22 (88.0)
Activity level (Knaus chronic health status score), *n* (%)		
Normal health status	8 (32.0)	11 (44.0)
Moderate activity limitation	15 (60.0)	13 (52.0)
Severe activity limitation due to chronic disease	2 (8.0)	1 (4.0)
Bedridden	0 (0.0)	0 (0.0)
SAPS II	65.0 [50.0; 71.0]	66.0 [51.0; 71.0]
History of any health condition	13 (52.0)	11 (44.0)
History of heart disease	10 (40.0)	7 (28.0)
History of pulmonary disease	5 (20.0)	6 (24.0)
Location at cardiac arrest		
Home	11 (44.0)	11 (44.0)
Public place	11 (44.0)	13 (52.0)
Hospital^b^	3 (12.0)	1 (4.0)
Bystander-witnessed cardiac arrest	23 (92.0)	25 (100.0)
Bystander performed CPR	21 (84)	20 (80.0)
Rhythm at cardiac arrest		
Ventricular fibrillation	20 (80)	24 (96.0)
Ventricular tachycardia	4 (16.0)	1 (4.0)
Cause of cardiac arrest		
Cardiac cause	23 (92.0)	24 (96.0)
Drowning	0 (0.0)	1 (4.0)
Asphyxia	2 (8.0)	0 (0.0)
Glasgow Coma Scale score at enrollment^c^	3 [3; 3]	3 [3; 5]
Corneal reflex present, n_1_ = 21, n_2_ = 17	15 (71.4)	11 (64.7)
Pupillary reflex present on the left, n_1_ = 25, n_2_ = 24	19 (76.0)	20 (83.3)
Pupillary reflex present on the right, n_1_ = 24, n_2_ = 24	18 (75.0)	18 (75.0)
ST-segment elevation myocardial infarction, n_1_ = 21, n_2_ = 19	11 (52.4)	14 (73.7)
Attempted coronary revascularization	11 (44.0)	18 (72.0)
Successful coronary revascularization	10 (40.0)	18 (72.0
Circulatory shock^d^	14 (56.0)	14 (56.0)
Serum pH, n_1_ = 25, n_2_ = 23	7.25 [7.18; 7.33]	7.32 [7.24; 7.36]
Lactate, mmol/L, n_1_ = 25, n_2_ = 23	2.5 [1.6; 4.3]	2.4 [1.1; 3.4]
No-flow duration^e^, minutes	0.0 [0.0; 2.0]	1.0 [0.0; 3.0]
Low-flow duration^f^, minutes	20.0 [15.0; 30.0]	20.0 [10.0; 30.0]
Epinephrine injection performed, n (%)	15 (60.0)	15 (60.0)
Epinephrine dose, mg, median [IQR], mg, n_1_ = 15, n_2_ = 15	3.0 [1.0; 4.0]	3.0 [1.0; 4.0]
Duration from cardiac arrest to randomization, hours	26.6 [25.0; 28.1]	26.5 [25.2; 27.6]
Body temperature at inclusion, °C	35.0 ± 1.0	35.0 ± 0.9
CAHP score^g^	145.9 [107.4; 167.1]	133.1 [93.8; 170.1]

The data are n (%) or median [25th; 75th percentiles]

^a^Charlson comorbidity index: Each comorbidity category is weighted from 1 to 6, based on the adjusted risk of mortality or resource use, and the sum of the weights produces the score for the patient. A score of zero indicates absence of known comorbidities. Higher scores indicate higher risks of death and greater resource use

^b^Four patients experienced cardiac arrest shortly after arrival at emergency rooms of community hospitals, achieved the ROSC, and were then immediately transferred to the study ICU

^c^Scores on the Glasgow Coma Scale can range from 3 to 15, with lower scores indicating worse consciousness impairment

^d^Circulatory shock was defined as a systolic blood pressure of less than 90 mmHg for at least 30 min or impaired end-organ perfusion (cool extremities, mottling, urine output < 30 mL per hour)

^e^No-flow duration was time from collapse to basic life-support initiation

^f^Low-flow duration was time from basic life-support initiation to return of spontaneous circulation

^g^The Cardiac Arrest Hospital Prognosis score is designed for the early stratification of patients admitted to the ICU after out-of-hospital cardiac arrest. Three risk groups are identified according to whether the score is ≤ 150, 150–200, or ≥ 200, with higher scores indicating a worse prognosis

### Targeted temperature management (TTM)

**Fig. 2:**
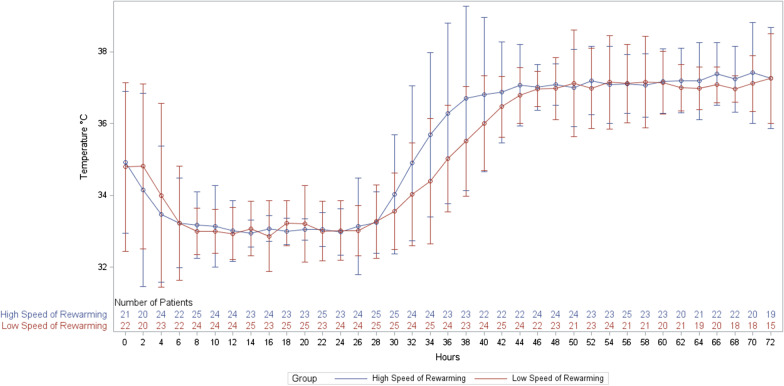
Body temperature during hypothermia and rewarming at 0.25 °C/h or 0.50 °C/h. The solid lines indicate the mean values and the bars the mean±2SDs (95% of the recorded values were within the bars). H0 is the time of ICU admission

The median [interquartile range] time from randomization to rewarming initiation was 4.5 [3.3–5.8] hours in the 0.25 °C/h group and 5.0 [3.3–5.4] hours in the 0.50 °C/h group. The median time from rewarming initiation and achievement of a body temperature of 37 °C was 16.5 [15.2–17.6] hours in the 0.25 °C/h group and 8.8 [8.2–10.2] hours in the 0.50 °C/h group. Figure 2 shows the body temperature time-course in each group and Additional file 3 the proportions of patients with fever in each group.

### Primary outcome

**Fig. 3:**
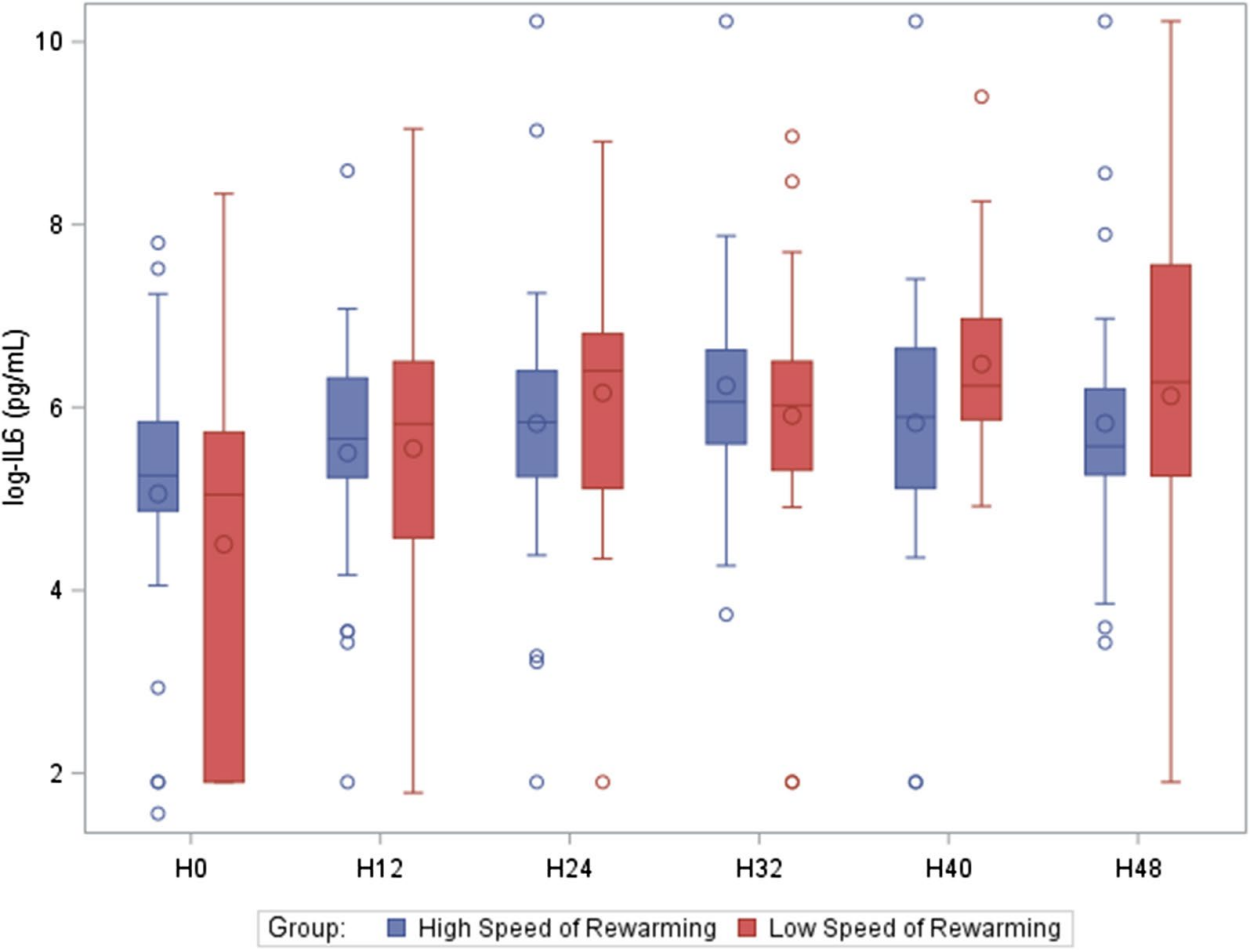
Time-course of serum interleukin-6 levels in the groups allocated at random to rewarming at 0.25 °C/h or 0.50 °C/h. The line inside the box indicates the median value, the bottom and top edges of the box indicate the intra-quartile range, and the circles show outliers. IL6 data were log-transformed to improve readability. H0 was the time of ICU admission

The median AUC for serum IL6 levels over time did not differ significantly between the 0.25 °C/h and 0.50 °C/h groups (12,389 pg/mL·h, [7256–37,200] vs. 8859 [6825–18,088]; *P* = 0.55). One patient died between H32 and H40 and was not included in this analysis given the absence of data for the H40 and H48 time points. The IL6 levels at each time point are reported in Additional file 4 and the IL6 level time-course in Fig. 3.

### Secondary outcomes

Except for IL2 at H40, the secondary-outcome biomarker levels were not significantly different between the two groups (Additional file 5). Median NF-L at H48 was 76.0 [25.5; 3074.0] pg/mL in the 0.25 °C/h group and 192 pg/mL [33.6; 4199.0] in the 0.50 °C/h group (*P* = 0.43). Median NSE at H48 was 27.2 [17.3; 83.8] ng/mL in the 0.25 °C/h group and 22.0 [16.1; 73.6] ng/mL in the 0.50 °C/h group (*P* = 0.77). Median NSE at H72 was 15.6 ng/mL [11.7; 58.2] and 14.1 [10.8; 112.1] ng/mL in the 0.25 and 0.50 °C/h groups, respectively (*P* = 0.62) (Additional file 6).

**Fig. 4:**
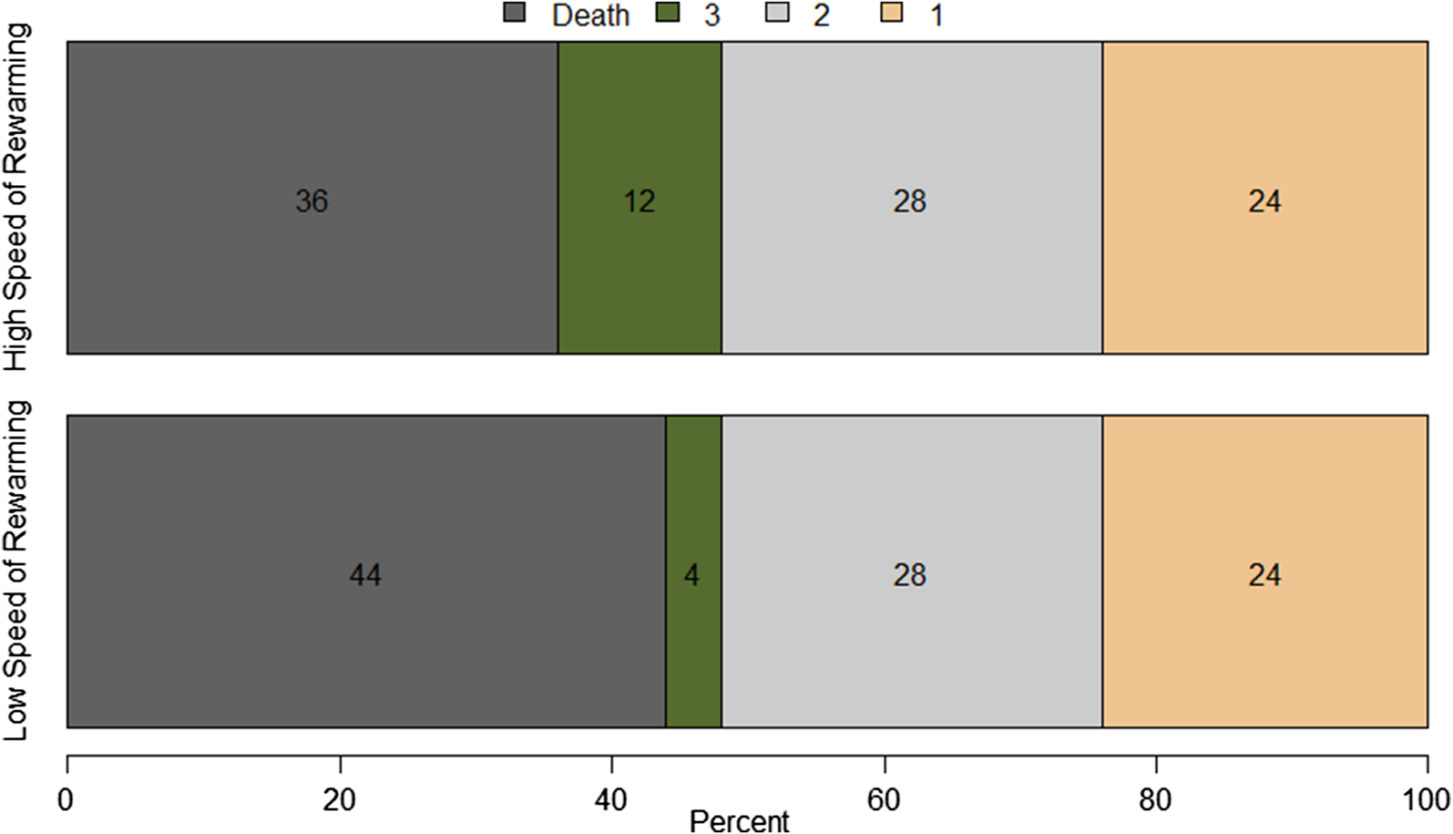
Distribution of Cerebral Performance Category scores in each group

On day 90 after randomization, the number of patients with a CPC score of 1 or 2 was 13 in both groups (52.0 vs. 52.0; 95% CI − 25.8; 25.8) (Fig. 4).

ICU mortality was not significantly different between the two groups, with a hazard ratio (HR) in the 0.25 °C/h group vs. the 0.50 °C/h group of 1.43 (95% CI 0.62; 3.32; *P* = 0.41). Neither was there any significant difference for in-hospital mortality (HR 1.43; 95% CI 0.62; 3.32; *P* = 0.41). ICU and hospital lengths of stay and MV duration are reported in Additional file 7.

### Tertiary and exploratory outcomes

Additional file 8 reports the proportions of patients with hypokalemia, hyperkalemia, hypoglycemia, and hyperglycemia. The proportions of patients with severe cardiac arrhythmias are given in Additional file 9 and changes over time in the cardiovascular Sequential Organ Failure Assessment score in Additional file 10.

## Discussion

In this single-center RCT, a slow rewarming rate of 0.25 °C/h after hypothermia at 33 °C in patients who were comatose after shockable OHCA did not significantly decrease serum IL6 levels compared to rewarming at 0.5 °C. The rewarming rate was not associated with significant differences in NF-L levels at H48 or functional outcome on day 90.

Whereas several RCTs have evaluated the role for TTM in patients who remain comatose after cardiac arrest [8–10, 22], none focused on the rewarming rate [5]. Observational data are conflicting. An ancillary analysis of data from a multicenter prospective cohort study found a significant association between very slow rewarming at a median of 0.045 °C/h from 34 to 36 °C and a CPC of 1 or 2 at hospital discharge [24]. In a retrospective study of 71 patients who received TTM with a target of 34 °C or lower after OHCA, the CPC score at 1 month was not significantly different between the groups rewarmed at 0.15 °C/h versus 0.25 °C/h [25]. Another retrospective cohort study found no significant difference in the Glasgow Outcome Scale score at 6 months between the groups with rewarming rates < 0.5 °C/h or ≥ 0.5 °C/h [26]. Differences in case-mix and in care delivered during TTM [15, 27], together with residual confounding, complicate the interpretation of these results. This probably explains why the 2021 update of guidelines for cardiac arrest make no recommendations about the rewarming rate [7].

Our primary outcome was a surrogate, i.e., serum IL6 levels. Current guidelines recommend day-90 functional outcome determined by blinded assessors as the primary outcome for trials of TTM after cardiac arrest. However, given the paucity of data on rewarming rates, for this pilot trial, we chose a primary outcome that required a far smaller sample size, to help in designing a future RCT with day-90 functional status as the primary outcome. Higher IL6 values have been reported to be associated with higher mortality, worse PCAS, and CPC of 3–5 at 12 months [11, 12]. In a clinical RCT of patients with OHCA, the IL6-receptor antagonist tocilizumab decreased systemic inflammation and myocardial injury without improving the functional outcome, which was not, however, the primary endpoint [28, 29]. Finally, the IL6 half-life of less than 1 h means that rapid changes in IL6 levels can be readily detected by serial assays [30].

In our study, NF-L levels 48 h after hypothermia initiation were not significantly different between the 0.25 °C/h and 0.50 °C/h rewarming groups. Recent data indicate a strong association between NF-L levels and the functional prognosis after cardiac arrest. A post hoc analysis of TTM trial data demonstrated better neuroprognostication performance of serum NF-L levels 24–72 h after cardiac arrest compared to many of the established parameters [31]. When added to a set of already available parameters (including NSE), NF-L 24–72 h after cardiac arrest improved the prognostic performance of a machine-learning algorithm [32]. NF-L was also an accurate and early predictor of long-term functional outcomes in the COMACARE trial in patients after OHCA [33].

The total hypothermia dose may deserve to be considered instead of the duration of induction, maintenance, and/or rewarming. If rewarming is slow, then the time spent in hypothermia is longer, amounting to a longer maintenance phase [5]. Retrospective data suggest that a higher hypothermia dose may be required to be effective in patients with longer no-flow times, i.e., worse anoxic-ischemic injuries [34]. In the recent HYPERION trial in patients seen after cardiac arrest in nonshockable rhythms, hypothermia at 33 °C for 24 h followed by rewarming at 0.22 °C/h improved day-90 functional outcomes compared to targeted normothermia [22, 35]. Nonetheless, studies assessing the links between the severity of hypoxic brain injury and the dose of hypothermia are conflicting [36–38]. Additionally, previous reports pooled patients managed with passive rewarming and active rewarming [8, 9]. Finally, the absence in many study reports of data on patient characteristics such as body mass index and on the cooling and warming devices used constitutes a major obstacle to meta-analyses.

Our trial has several limitations. Patient recruitment occurred at a single center. However, this pilot trial allowed us to confirm the feasibility of delayed randomization [39] of the rewarming rate to avoid including patients who died during hypothermia induction or maintenance and who consequently could not receive the allocated rewarming rate. We included only patients with OHCA in a shockable rhythm, although patients with a nonshockable rhythm may benefit the most from high doses of hypothermia [22]. Finally, we used a surrogate as the primary outcome. A larger sample would have been needed to compare functional outcomes, such the modified Rankin scale score, as recommended by COSCA guidelines [40]. Finally, the results of the recent TTM2 trial [41] may lead to a reduction in the use of targeted hypothermia (between 32 and 34 °C), since no survival advantage was found. Nevertheless, our results may help to determine the optimal rewarming strategy (including the prevention of fever).

## Conclusion

In conclusion, compared to rewarming at 0.5 °C/H, rewarming at 0.25 °C/h after hypothermia at 33 °C for 24 h did not decrease serum IL6 levels in patients who remained comatose after cardiac arrest in a shockable rhythm. Further RCTs are needed to better define the optimal TTM modalities for patients with cardiac arrest.

## Supplementary Information

**Additional file 1**: Randomization window and blood sampling

**Additional file 2**: Baseline characteristics of patients assessed for eligibility

**Additional file 3**: Proportion of febrile patients

**Additional file 4**: Time-course of serum IL6 levels

**Additional file 5**: Time-course of the other biomarkers

**Additional file 6**: Comparison of NSE on days 2 and 3

**Additional file 7**: Outcomes

**Additional file 8**: Proportions of patients with metabolic disorders

**Additional file 9**: Proportions of patients with severe cardiac arrhythmias

**Additional file 10**: Evolution of cardiovascular of SOFA score in both groups

## Data Availability

The study data will be available from the corresponding author upon reasonable request.
