# Supplementary figures and images for: Impact of rewarming rate on interleukin-6 levels in patients with shockable cardiac arrest receiving targeted temperature management at 33 °C: the ISOCRATE pilot randomized controlled trial
Source: Crit Care. 2021 Dec 17;25:434. doi: 10.1186/s13054-021-03842-9 (PMC8680374; doi:10.1186/s13054-021-03842-9)

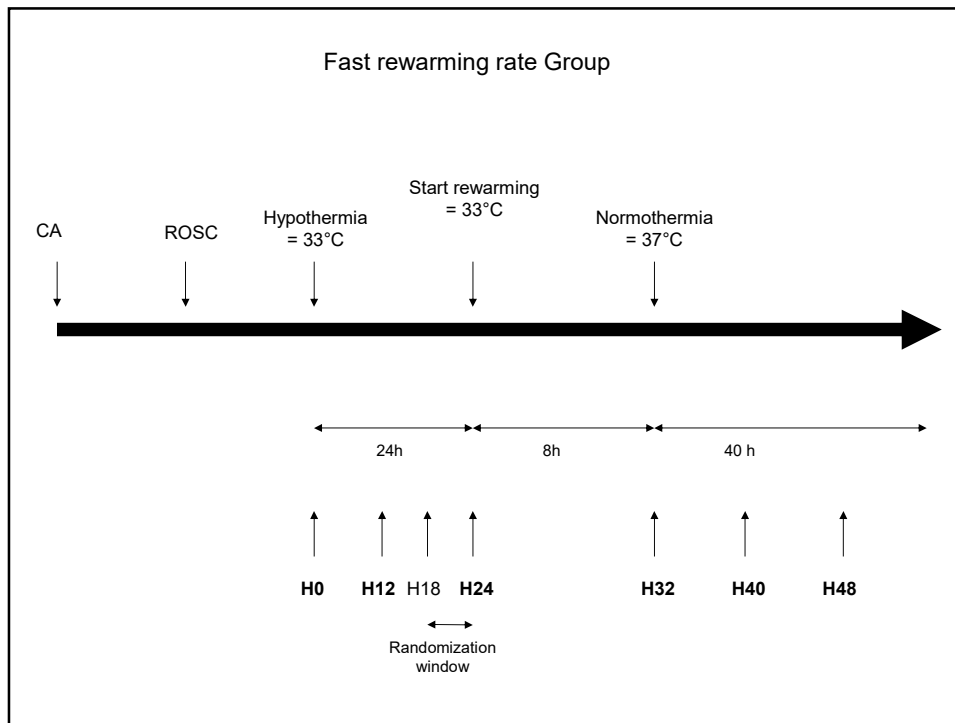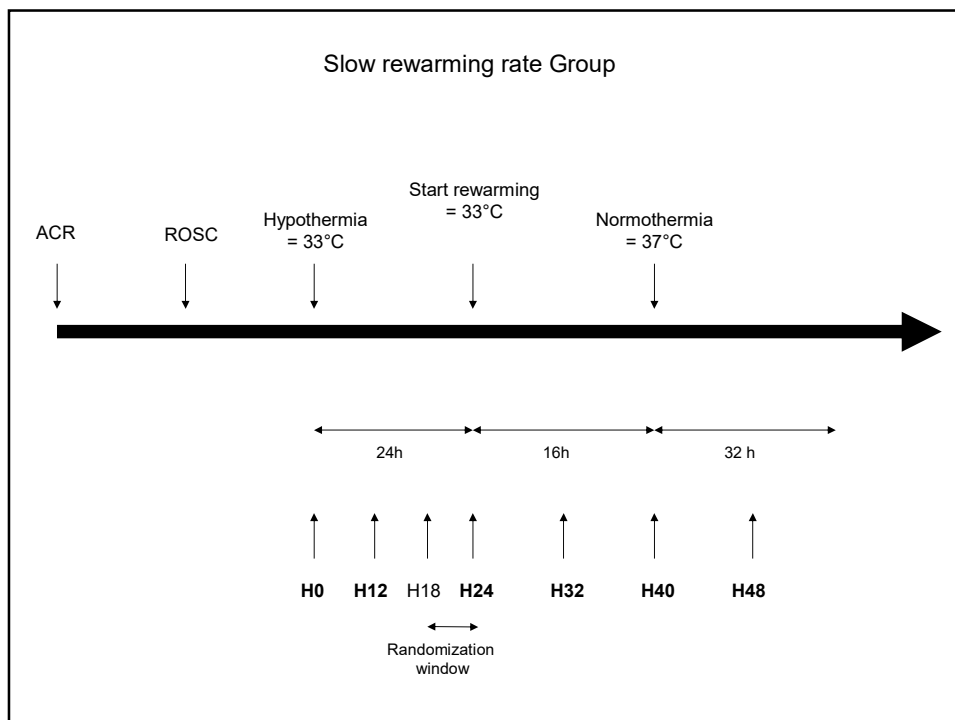

Supplement: Supplementary file 1 — Additional file 1: Randomization window and blood sampling [file 13054_2021_3842_MOESM1_ESM.pdf]

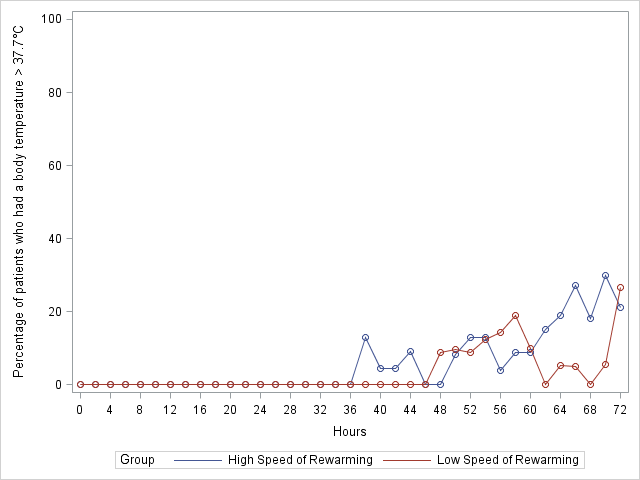


H0 was the time at ICU admission.

Supplement: Supplementary file 3 — Additional file 3: Proportion of febrile patients [file 13054_2021_3842_MOESM3_ESM.docx]
