# Supplementary material for: Impact of rewarming rate on interleukin-6 levels in patients with shockable cardiac arrest receiving targeted temperature management at 33 °C: the ISOCRATE pilot randomized controlled trial
Source: Crit Care. 2021 Dec 17;25:434. doi: 10.1186/s13054-021-03842-9 (PMC8680374; doi:10.1186/s13054-021-03842-9)
Supplement: Supplementary file 2 — Additional file 2: Baseline characteristics of patients assessed for eligibility [file 13054_2021_3842_MOESM2_ESM.docx]

|  | **Assessed for eligibility but not included or randomized**  **(n_1_=8)** | **Randomized**  **(n_2_=50)** |
| --- | --- | --- |
| Age, median [IQR], years | 61.0 [54.0 ; 67.3] | 61.9 [48.7 ; 72.2] |
| Male sex, n (%) | 5 (62.5) | 41 (82.0) |
| Rhythm at cardiac arrest, n (%)  *Ventricular fibrillation*  *Ventricular tachycardia* | 8 (100.0)  0 (0.0) | 44 (89.8)  5 (10.2) |
| Glasgow Coma Scale score, median [IQR] | 3.0 [3.0 ; 3.0] | 3.0 [3.0 ; 6.0] |
| No-flow time, median [IQR], minutes | 2.0 [2.0 ; 3.0] | 0.0 [0.0 ; 3.0] |
| Low-flow time, median [IQR], minutes | 15.0 [10.0 ; 30.0] | 20.0 [12.0 ; 30.0] |

**Additional File 2**: Baseline characteristics of patients assessed for eligibility
