# Supplementary material for: Impact of rewarming rate on interleukin-6 levels in patients with shockable cardiac arrest receiving targeted temperature management at 33 °C: the ISOCRATE pilot randomized controlled trial
Source: Crit Care. 2021 Dec 17;25:434. doi: 10.1186/s13054-021-03842-9 (PMC8680374; doi:10.1186/s13054-021-03842-9)
Supplement: Supplementary file 4 — Additional file 4: Time-course of serum IL6 levels [file 13054_2021_3842_MOESM4_ESM.docx]

|  | **Interleukin 6 level** (pg/mL), median [IQR] | |  |
| --- | --- | --- | --- |
| **Time point** | **0.25°C/h**  **(n1=25)** | **0.50°C/h**  **(n2=25)** | ***P* value**  **Wilcoxon’s test** |
| H0, n_1_=25, n_2_=25 | 155.8 [6.7 ; 306.6] | 191.4 [130.7 ; 343.9] |  |
| H12, n_1_=24^a^, n_2_=25 | 337.3 [97.7 ; 665.0] | 285.9 [187.6 ; 555.8] |  |
| H24, n_1_=25, n_2_=25 | 600.9 [166.4 ; 900.7] | 342.9 [188.8 ; 600.1] | 0.36 |
| H32, n_1_=24^a^, n_2_=25 | 412.3 [202.9 ; 667.2] | 427.3 [270.6 ; 754.3] | 0.51 |
| H40, n_1_=24^b^, n_2_=25 | 511.7 [352.8 ; 1069.2] | 362.9 [166.7 ; 767.2] | 0.12 |
| H48, n_1_=24^b^, n_2_=25 | 532.1 [191.4 ; 1949.9] | 263.4 [192.8 ; 493.5] | 0.21 |

^a^No data were available for the H12 and H32 time points in 1 patient.

^b^One patient died between H32 and H40.

IQR: interquartile range
