# Supplementary material for: Impact of rewarming rate on interleukin-6 levels in patients with shockable cardiac arrest receiving targeted temperature management at 33 °C: the ISOCRATE pilot randomized controlled trial
Source: Crit Care. 2021 Dec 17;25:434. doi: 10.1186/s13054-021-03842-9 (PMC8680374; doi:10.1186/s13054-021-03842-9)
Supplement: Supplementary file 5 — Additional file 5: Time-course of the other biomarkers [file 13054_2021_3842_MOESM5_ESM.docx]

**Additional File 5: Interleukin(IL)-2, IL4, IL8, IL10, granulocyte-macrophage colony-stimulating factor (GM-CSF), tumor necrosis factor (TNF)α, C-reactive protein, and procalcitonin at each of six timepoints during the first 48 hours after reaching the target temperature (33°C) in the two rewarming-rate groups**

**eTable 3a: Interleukin 2** values were dichotomized on the detection threshold; the numbers and percentages in the table are those of patients with levels below the threshold.

| **Hours** | **0.25°C/h**  **(n1=25)** | **0.50°C/h**  **(n2=25)** | ***P***  **Fisher’s Test** |
| --- | --- | --- | --- |
| H0  n_1_=25, n_2_=25 | 21 (84) | 22.0 (88) |  |
| H12  n_1_=24, n_2_=25 | 23 (96) | 24.0 (96) |  |
| H24  n_1_=25, n_2_=25 | 20 (80) | 21.0 (84) | 1.00 |
| H32  n_1_=24, n_2_=25 | 20 (83) | 21.0 (84) | 1.00 |
| H40  n_1_=24, n_2_=25 | 18 (75) | 24.0 (96) | 0.0488 |
| H48  n_1_=24, n_2_=25 | 19 (79) | 22.0 (88) | 0.46 |

**eTable 3b: Interleukin 4** values were dichotomized on the detection threshold; the numbers and percentages in the table are those of patients with levels below the threshold.

| **Hours** | **0.25°C/h**  **(n1=25)** | **0.50°C/h**  **(n2=25)** | ***P* value**  **Fisher’s Test** |
| --- | --- | --- | --- |
| H0,  n_1_=25, n_2_=25 | 23 (92) | 22.0 (88) |  |
| H12,  n_1_=24, n_2_=25 | 24 (100) | 22.0 (88) |  |
| H24,  n_1_=25, n_2_=25 | 23 (92) | 23.0 (92) | 1.00 |
| H32,  n_1_=24, n_2_=25 | 24 (100) | 23.0 (92) | 0.49 |
| H40,  n_1_=24, n_2_=25 | 22 (92) | 24.0 (96) | 0.61 |
| H48,  n_1_=24, n_2_=25 | 23 (96) | 23.0 (92) | 1.00 |

**eTable 3c: Interleukin 8** values were dichotomized on the detection threshold; the numbers and percentages in the table are those of patients with levels below the threshold.

| **Hours** | **0.25°C/h**  **(n1=25)** | **0.50°C/h**  **(n2=25)** | ***P* value**  **Fisher’s Test** |
| --- | --- | --- | --- |
| H0,  n_1_=25, n_2_=25 | 19 (76) | 20.0 (80) |  |
| H12,  n_1_=24, n_2_=25 | 21 (87) | 22.0 (88) |  |
| H24,  n_1_=25, n_2_=25 | 22 (88) | 21.0 (84) | 1.00 |
| H32,  n_1_=24, n_2_=25 | 20 (83) | 21.0 (84) | 1.00 |
| H40,  n_1_=24, n_2_=25 | 21 (87) | 24.0 (96) | 0.35 |
| H48,  n_1_=24, n_2_=25 | 21 (87) | 22.0 (88) | 1.00 |

**eTable 3d: Interleukin 10** values (median [interquartile range] in the two groups

| **Hours** | **0.25°C/h**  **(n1=25)** | **0.50°C/h**  **(n2=25)** | ***P* value**  **Wilcoxon’s test** |
| --- | --- | --- | --- |
| H0,  n_1_=25, n_2_=25 | 7.4 [2.7 ; 23.9] | 7.4 [4.1 ; 24.2] |  |
| H12,  n_1_=24, n_2_=25 | 4.0 [0.0 ; 6.2] | 4.2 [3.1 ; 11.4] |  |
| H24,  n_1_=25, n_2_=25 | 5.1 [4.1 ; 7.8] | 6.0 [3.1 ; 9.9] | 0.63 |
| H32,  n_1_=24, n_2_=25 | 5.7 [4.5 ; 10.2] | 11.0 [6.2 ; 15.6] | 0.02 |
| H40,  n_1_=24, n_2_=25 | 10.2 [6.9 ; 22.2] | 6.9 [4.3 ; 12.0] | 0.13 |
| H48,  n_1_=24, n_2_=25 | 9.5 [3.7 ; 15.0] | 5.2 [3.1 ; 9.7] | 0.19 |

**eTable 3e: GM-CSF** values were dichotomized on the detection threshold; the numbers and percentages in the table are those of patients with levels below the threshold.

| **Hours** | **0.25°C/h**  **(n1=25)** | **0.50°C/h**  **(n2=25)** | ***P***  **Fisher’s Test** |
| --- | --- | --- | --- |
| H0,  n_1_=25, n_2_=25 | 22 (88) | 23 (92) |  |
| H12,  n_1_=24, n_2_=25 | 24 (100) | 25 (100) |  |
| H24,  n_1_=25, n_2_=25 | 24 (96) | 25 (100) | 1.00 |
| H32,  n_1_=24, n_2_=25 | 23 (96) | 24 (96) | 1.00 |
| H40,  n_1_=24, n_2_=25 | 23 (96) | 25 (100) | 0.49 |
| H48,  n_1_=24, n_2_=25 | 23 (96) | 23 (92) | 1.00 |

**eTable 3f: TNFα** values were dichotomized on the detection threshold; the numbers and percentages in the table are those of patients with levels below the threshold.

| **Hours** | **0.25°C/h**  **(n1=25)** | **0.50°C/h**  **(n2=25)** | ***P* value**  **Fisher’s Test** |
| --- | --- | --- | --- |
| H0,  n_1_=25, n_2_=25 | 21 (84) | 24 (96) |  |
| H12,  n_1_=24, n_2_=25 | 22 (91) | 24 (96) |  |
| H24,  n_1_=25, n_2_=25 | 22 (88) | 23 (92) | 1.00 |
| H32,  n_1_=24, n_2_=25 | 22 (92) | 22 (88) | 1.00 |
| H40,  n_1_=24, n_2_=25 | 20 (83) | 22 (88) | 0.70 |
| H48,  n_1_=24, n_2_=25 | 18 (75) | 23 (92) | 0.14 |

**eTable 3g: CRP** values during targeted temperature management and the next 7 days; the data are median [IQR].

| **Hours** | **0.25°C/h**  **(n1=25)** | **0.50°C/h**  **(n2=25)** | ***P* value**  **Wilcoxon’s test** |
| --- | --- | --- | --- |
| Inclusion,  n_1_=21, n_2_=17 | 2.0 [1.0 ; 5.0] | 3.0 [1.0 ; 6.0] |  |
| At 33°C,  n_1_=22, n_2_=20 | 4.5 [2.0 ; 11.0] | 5.5 [3.0 ; 18.0] |  |
| Day 1,  n_1_=25, n_2_=21 | 19.0 [10.0 ; 44.0] | 30.0 [16.0 ; 51.0] | 0.11 |
| Day 2,  n_1_=23, n_2_=23 | 119.0 [80.0 ; 187.0] | 159.0 [116.0 ; 213.0] | 0.21 |
| Day 3,  n_1_=20, n_2_=24 | 207.0 [95.0 ; 273.0] | 212.5 [133.0 ; 271.0] | 0.79 |
| Day 4,  n_1_=15, n_2_=21 | 195.0 [129.0 ; 267.0] | 152.0 [90.0 ; 244.0] | 0.50 |
| Day 5,  n_1_=17, n_2_=18 | 160.0 [98.0 ; 262.0] | 163.5 [84.0 ; 232.0] | 1.00 |
| Day 6,  n_1_=12, n_2_=16 | 124.0 [99.5 ; 180.0] | 144.0 [53.5 ; 224.0] | 0.85 |
| Day 7,  n_1_=13, n_2_=14 | 74.0 [66.0 ; 132.0] | 161.0 [67.0 ; 222.0] | 0.16 |

**eTable 3h: Procalcitonin**

The interaction term indicated that group allocation did not influence the time-course of serum procalcitonin levels (*P*=0.72). Procalcitonin values were normally distributed and were compared using linear mixed regression.

| **Effect** | **Value and 95%CI** | ***P* value** |
| --- | --- | --- |
| Intercept | -0.596 [-1.276 ; 0.083] | 0.0829 |
| Days | 0.037 [-0.072 ; 0.146] | 0.5025 |
| Group | 0.049 [-0.906 ; 1.005] | 0.9190 |
| Interaction between group and days | 0.028 [-0.129 ; 0.184] | 0.7258 |
