# Supplementary material for: Impact of rewarming rate on interleukin-6 levels in patients with shockable cardiac arrest receiving targeted temperature management at 33 °C: the ISOCRATE pilot randomized controlled trial
Source: Crit Care. 2021 Dec 17;25:434. doi: 10.1186/s13054-021-03842-9 (PMC8680374; doi:10.1186/s13054-021-03842-9)
Supplement: Supplementary file 6 — Additional file 6: Comparison of NSE on days 2 and 3 [file 13054_2021_3842_MOESM6_ESM.docx]

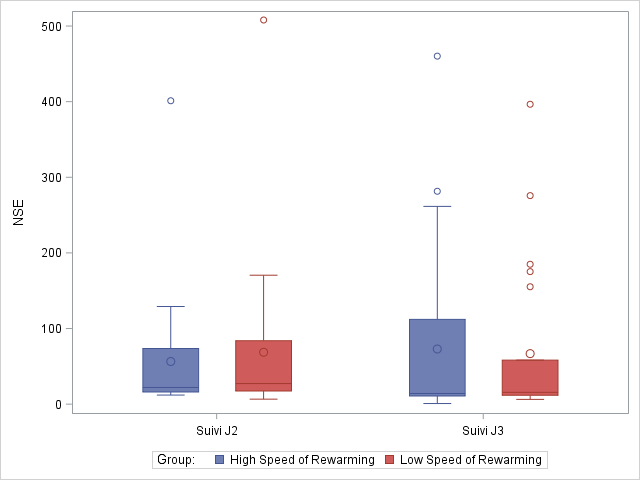


Day 3

Day 2

The line inside the box indicates the median value, the bottom and top edges of the box indicate the intra-quartile range, and the circles show outliers.
