# Supplementary material for: Impact of rewarming rate on interleukin-6 levels in patients with shockable cardiac arrest receiving targeted temperature management at 33 °C: the ISOCRATE pilot randomized controlled trial
Source: Crit Care. 2021 Dec 17;25:434. doi: 10.1186/s13054-021-03842-9 (PMC8680374; doi:10.1186/s13054-021-03842-9)
Supplement: Supplementary file 7 — Additional file 7: Outcomes [file 13054_2021_3842_MOESM7_ESM.docx]

**Additional File 7:** Mortality, ICU stay length, and duration of endotracheal mechanical ventilation

|  | **0.25°C/h group**  **(n=25)** | **0.50°C/h group**  **(n=25)** |
| --- | --- | --- |
| **Patients who died, n** | 11 | 9 |
| ICU length of stay, median [IQR] | 3.0 [2.0 ; 6.0] | 5.0 [4.0 ; 8.0] |
| Mechanical ventilation duration, median [IQR] | 3.0 [2.0 ; 6.0] | 5.0 [4.0 ; 8.0] |
|  |  |  |
| **Survivors, n** | 14 | 16 |
| ICU length of stay, median [IQR] | 6.5 [4.0 ; 8.0] | 6.5 [4.5 ; 10.5] |
| Mechanical ventilation duration, median [IQR] | 4.5 [2.0 ; 7.0] | 4.5 [4.0 ; 8.5] |
