# Supplementary material for: Impact of rewarming rate on interleukin-6 levels in patients with shockable cardiac arrest receiving targeted temperature management at 33 °C: the ISOCRATE pilot randomized controlled trial
Source: Crit Care. 2021 Dec 17;25:434. doi: 10.1186/s13054-021-03842-9 (PMC8680374; doi:10.1186/s13054-021-03842-9)
Supplement: Supplementary file 8 — Additional file 8: Proportions of patients with metabolic disorders [file 13054_2021_3842_MOESM8_ESM.docx]

**Additional File 8:** Proportions of patients with metabolic disorders

|  | High rewarming rate  (n = 25) | Low rewarming rate  (n = 25) |
| --- | --- | --- |
| Patients with hypokalemia, n (%) | 8.0 | 20.0 |
| Patients with hyperkalemia, n (%) | 16.0 | 48.0 |
| Patients with hypoglycemia, n (%) | 0.0 | 0.0 |
| Patients with hyperglycemia, n (%) | 64.0 | 68.0 |

Hypokalemia was defined as serum potassium lower than 3 mEq/L and hyperkalemia as serum potassium higher than 5 mEq/L. Serum potassium alterations were monitored during the first 3 days in the ICU.

Hypoglycemia was defined as blood glucose lower than 40 mg/dL and hyperglycemia as blood glucose higher than 180 mg/dL. Blood glucose alterations were monitored during the first 7 days in the ICU.
