# Supplementary material for: Impact of rewarming rate on interleukin-6 levels in patients with shockable cardiac arrest receiving targeted temperature management at 33 °C: the ISOCRATE pilot randomized controlled trial
Source: Crit Care. 2021 Dec 17;25:434. doi: 10.1186/s13054-021-03842-9 (PMC8680374; doi:10.1186/s13054-021-03842-9)
Supplement: Supplementary file 9 — Additional file 9: Proportions of patients with severe cardiac arrhythmias [file 13054_2021_3842_MOESM9_ESM.docx]

**Additional File 9.** Proportion of patients with severe cardiac arrhythmia

|  | **Severe cardiac arrhythmia** | |
| --- | --- | --- |
|  | **Low rewarming rate**  **(n1=25)** | **High rewarming rate**  **(n2=25)** |
| Day 2, *n_1_=25, n_2_=25* | 1 (4.0) | 1 (4.0) |
| Day 3, *n_1_=23, n_2_=25* | 2 (8.7) | 2 (8.0) |
| Day 4, *n_1_=19, n_2_=23* | 2 (10.5) | 1 (4.3) |
| Day 5, *n_1_=17, n_2_=21* | 1 (5.9) | 1 (4.8) |
| Day 6, *n_1_=14, n_2_=20* | 1 (7.1) | 0 (0.0) |
| Day 7, *n_1_=13, n_2_=14* | 1 (7.7) | 0 (0.0) |
