# Supplementary material for: Impact of rewarming rate on interleukin-6 levels in patients with shockable cardiac arrest receiving targeted temperature management at 33 °C: the ISOCRATE pilot randomized controlled trial
Source: Crit Care. 2021 Dec 17;25:434. doi: 10.1186/s13054-021-03842-9 (PMC8680374; doi:10.1186/s13054-021-03842-9)
Supplement: Supplementary file 10 — Additional file 10: Evolution of cardiovascular of SOFA score in both groups [file 13054_2021_3842_MOESM10_ESM.docx]

**Additional File 9.** Time-course of the cardiovascular SOFA score in both groups

|  | **Cardiovascular SOFA score** | |
| --- | --- | --- |
|  | **Low rewarming rate**  **(n1=25)** | **High rewarming rate**  **(n2=25)** |
| Day 2, *n_1_=25, n_2_=25* | 4.0 [4.0 ; 4.0] | 4.0 [1.0 ; 4.0] |
| Day 3, *n_1_=23, n_2_=25* | 4.0 [1.0 ; 4.0] | 1.0 [1.0 ; 4.0] |
| Day 4, *n_1_=19, n_2_=23* | 1.0 [0.0 ; 3.0] | 1.0 [0.0 ; 3.0] |
| Day 5, *n_1_=17, n_2_=21* | 0.0 [0.0 ; 1.0] | 1.0 [0.0 ; 1.0] |
| Day 6, *n_1_=14, n_2_=19* | 1.0 [0.0 ; 1.0] | 1.0 [0.0 ; 1.0] |
| Day 7, *n_1_=13, n_2_=13* | 1.0 [0.0 ; 1.0] | 0.0 [0.0 ; 1.0] |
